# Earthquakes, attributions, and psychopathology: a study in a rural community

**DOI:** 10.1080/20008198.2019.1661813

**Published:** 2019-09-05

**Authors:** Alessandro Massazza, Helene Joffe, Chris R. Brewin

**Affiliations:** Research Department of Clinical, Educational and Health Psychology, University College London, London, UK

**Keywords:** Disaster survivors, posttraumatic stress disorder, psychopathology, attributions, blame, cause, sobrevivientes de desastre, Trastorno por estrés postraumático, psicopatología, atribuciones, culpa, causa, 灾难幸存者, 创伤后应激障碍, 精神病症, 归因, 责任, 原因

## Abstract

**Background**: Attributions of both cause and blame form part of the diagnostic criteria for PTSD in DSM-5. Most work on attributions and psychopathology has focused on survivors of interpersonal violence and the two types of attribution have not been investigated together in natural disaster contexts. Previous work has identified that attributions to God’s role may be associated with survivors’ mental health following disasters. We studied the relation between attributions to God and other actors/entities in a rural community with high levels of religiosity that had suffered extensive damage and loss of life due to a series of earthquakes.

**Methods**: A sample of survivors (*N* = 127) was assessed for degree of earthquake exposure, resource loss, attributions of cause and blame for the earthquake damage, and psychopathology three months after a series of major earthquakes in Italy.

**Results**: Nature and chance were associated with higher cause than blame attributions whereas the State, the municipality, building firms, and the mafia were associated with higher blame than cause attributions. Additionally, both cause and blame attributions towards God and chance were positively correlated with PTSD and psychological distress symptoms. These associations remained significant while controlling for degree of earthquake exposure, resource loss, gender, age, and education.

**Conclusion**: The current study supports the role played by cognitions about the cause of traumatic events, as introduced into the PTSD diagnosis in DSM-5, and extends this to blame of other entities such as God and chance following disasters.

## HIGHLIGHTS

• Attributions towards God and chance by earthquake survivors were positively associated with PTSD symptoms and psychological distress.• Survivors distinguished between cause and blame attributions.• Supporting evidence is provided for DSM-5 D3 criterion for PTSD.

## Abstract (es)

**Antecedentes**: las atribuciones de causa y culpa forman parte de los criterios diagnósticos para el TEPT en el DSM-5. La mayor parte del trabajo sobre atribuciones y psicopatología se ha centrado en los sobrevivientes de violencia interpersonal y los dos tipos de atribución no se han investigado juntos en contextos de desastres naturales. Trabajos previos han identificado que las atribuciones al papel de Dios pueden estar asociadas con la salud mental de los sobrevivientes luego de los desastres. Estudiamos la relación entre las atribuciones a Dios y otros actores/entidades en una comunidad rural con altos niveles de religiosidad que habían sufrido daños extensos y pérdida de vidas debido a una serie de terremotos.

**Métodos**: Una muestra de sobrevivientes (N = 127) fue evaluada respecto del grado de exposición al terremoto, la pérdida de recursos, las atribuciones de causa y culpa del daño del terremoto y la psicopatología tres meses después de una serie de grandes terremotos en Italia.

**Resultados**: la naturaleza y el azar se asociaron con una mayor atribución de causa que culpa, mientras que el Estado, el municipio, las empresas de construcción y la mafia se asociaron con una mayor atribución de culpa que causa. Además, las atribuciones de causa y culpa hacia Dios y el azar se correlacionaron positivamente con TEPT y síntomas de alteración psicológica. Estas asociaciones se mantuvieron significativas al controlar por grado de exposición al terremoto, pérdida de recursos, género, edad y educación.

**Conclusión**: El estudio actual respalda el rol que juegan las cogniciones sobre la causa de eventos traumáticos, como se introdujo en el DSM-5 para el diagnóstico de TEPT, y extiende esto a la culpabilización de otras entidades como Dios y el azar luego de los desastres.

## Abstract (zh)

**背景：**对原因和责任的归因是DSM-5中PTSD诊断标准的一部分。大多数关于归因和精神病症的工作都将关注点放在了人际暴力的幸存者身上，并未在自然灾害背景下同时考查过这两种归因。此前的研究已经发现，在灾难发生后，归因于上帝可能与幸存者的心理健康有关。在一个有高度宗教信仰、因一系列地震遭受了重大破坏和生命损失的乡村社区，我们研究了归因于上帝与其他行为主体/实体之间的关系。

**方法：**在意大利一系列大地震发生三个月后，对127名幸存者进行了地震暴露程度、资源损失、对震害的原因和责任归因以及精神病症的评估。

**结果：**自然和意外与原因归因的相关高于责任归因，而国家、自治区、建筑公司和黑手党与责任归因的相关高于原因归因。此外，对上帝和意外的原因和责任归因都与创伤后应激障碍和心理困扰症状呈正相关。在控制了地震暴露程度、资源损失、性别、年龄和教育程度后，这些关联仍然显著。

**结论：**本研究支持对创伤事件原因的认知发挥的作用，正如其被DSM-5中的创伤后应激障碍诊断引入，并扩展为灾后对其他实体（如上帝和意外）的归咎。

## 1. Introduction

### 1.1. Earthquakes, psychopathology and attributions

Earthquakes can represent highly traumatic events for the exposed populations due to their suddenness and unpredictability, their capacity to destroy large areas, which can result in extensive material and human loss, and the presence of aftershocks following the primary quake (McCaughey, Hoffman, & Llewellyn, 1995). The first meta-analysis on posttraumatic stress disorder (PTSD) following earthquakes reported a prevalence of 29% for clinical PTSD levels up to 9 months following earthquakes in a sample of 76,101 survivors (Dai et al., 2016). Findings from the most studied earthquake in Italy, the 2006 L’Aquila earthquake, support those results with reported rates of probable PTSD diagnoses ranging from 35% to 40% of the exposed population (Stratta, Rossetti, Di Michele, & Rossi, 2016).

Among the psychological variables that have been identified as risk factors for the development and maintenance of PTSD, the process of attribution, that is, the construction of explanations for the traumatic event, has been consistently found to be important (Feiring & Cleland, 2007; Joseph, Yule, & Williams, 1993). Indeed, DSM-5 has recently added to the PTSD diagnosis the D3 symptom of ‘*persistent, distorted cognitions about the cause or consequences of the traumatic event(s) that lead the individual to blame himself/herself or others*’ (APA, 2013, p. 272).

However, most of the research on the relationship between attributions and PTSD has focused on the positive association between internal attributions, or self-blame, and PTSD among survivors of interpersonal trauma such as rape and assault (Massad & Hulsey, 2006). Less research has focused on the attributions made by survivors of natural disasters, so that for this group there is less of an evidence base for the new DSM-5 symptom concerning perceptions of cause and blame.

### 1.2. Attributions and psychopathology following disaster

Within the literature addressing the relationship between attributions and psychopathology following disaster, one early study reported higher levels of distress among adult survivors who blamed themselves following a lightning strike (Dollinger, 1986). Another study also reported that individuals who blamed themselves following a flood presented with higher levels of somatization (Solomon, Regier, & Burke, 1989). Similarly, a study reported that self-blame was positively associated with more severe depressive symptoms among students five months following a hurricane (Jeney-Gammon, Daugherty, Finch, Belter, & Foster, 1993). Further support for the positive association between internal, stable, and global attributions and depressive symptoms is provided by a study among survivors of the 1994 Northridge earthquake (Greening, Stoppelbein, & Docter, 2002). More recently, similar associations were found among survivors of the 2004 Indian Ocean tsunami (Banford, Wickrama, & Ketring, 2014; Levy, Slade, & Ranasinghe, 2009) and of a tornado in Oklahoma (Lack & Sullivan, 2008).

Furthermore attributing the event to God appears to be a rather common practice across different cultures following disasters. Among 1,253 South East Anatolian women who had survived the 2003 Bingöl earthquake, the majority of them explained the earthquake as caused by the will of God (51%) and by nature (41%), while human responsibility was considered less accountable (7%) (Sezgin & Punamäki, 2012). Individuals who believed the earthquake was a result of God’s decision reported higher levels of depression and somatization. Similar associations between assigning responsibility to God and increased levels of psychopathology have been found among survivors of the 2005 Kashmir earthquake (Feder et al., 2013) and of floods in the US (Smith, Pargament, Brant, & Oliver, 2000). Importantly the impact of religious attributions on psychopathology following disaster may be different according to the type of religion and the characteristics of the worshipped entity (Aten et al., 2019; Park, 2016).

### 1.3. The distinction between attributions of cause and attributions of blame

Studies of people’s explanations have often been oblivious to the distinction between attributions of cause and attributions of blame (Shaver & Drown, 1986). While causal attributions consist of beliefs about what logically led to a specific outcome, blame attributions are concerned with whether this happened in an immoral way or not (Malle, Guglielmo, & Monroe, 2014). Empirical work has shown that individuals do make this distinction in practice, for example following injuries in industrial accidents (Brewin, 1984). In addition, whether the attribution concerns cause or blame can have significant clinical consequences. According to Brewin (1984) while an internally directed causal attribution can lead to an increased perception of future control and therefore to better adjustment, an internally directed blame attribution is likely to carry with it negative emotions such as guilt and shame that hinder psychological wellbeing. Unlike perceived causation, blame is intrinsically tied up with morality and holds as much social as cognitive salience. Indeed, according to Malle *et al*.’s (2014) theory, blame is entrenched in the realm of social cognition, as it is always a judgement based on a series of mental states involving intentionality, foreseeability, obligation, and control over the situation (Alicke, 2000; Lagnado & Channon, 2008).

### 1.4. Limitations in the literature and hypotheses

Therefore, existing research on the link between attributions and psychopathology contains several limitations. Firstly, most of the literature has focused on internally directed blame attributions following interpersonal violence with less attention to other types of traumas. Secondly, even within the disaster literature, the attributions under scrutiny are often limited to simple distinctions between attributions towards the self and attributions towards others. This reflects attributional research following interpersonal violence. However, it is likely that in a disaster setting many more agents are at play. Moreover, according to Hall, French, and Marteau (2003), one of the reasons for the inconsistent findings in their systematic review of attributions and psychopathology is the lack of consistency in the terminology used, and, in particular, the inability to distinguish attributions of cause from attributions of blame (Shaver & Drown, 1986). Finally, despite the importance of gender for PTSD (Olff, 2017), relatively little attention has been paid in the earthquake literature to this, to other demographic factors such as age and education, or to the potential confounding effects of degree of trauma exposure and resource loss.

The current study attempts to overcome the limitations in the existing literature by addressing the following research questions. Firstly, we explore what entities were deemed responsible for the earthquake’s damage by survivors and whether attributions of cause and blame differ for the same entity. Secondly we investigate the association between attributions of cause and blame towards different entities and levels of PTSD and psychological distress. Finally, we explore the effect of earthquake exposure, resource loss, and demographic variables in the relationship between attributions and psychopathology. Based on past research we advance three hypotheses:

Hypothesis 1: Participants will distinguish between attributions of cause and attributions of blame.

Hypothesis 2: Attributions to God will be associated with greater rates of psychopathology.

Hypothesis 3: The relationship between God attributions and psychopathology will remain significant after controlling for resource loss, earthquake exposure, and demographic variables.

### 1.5. The 2016–2017 central Italy earthquake sequence

At 3.36 am on the 24th of August 2016 a 6.0 Mw earthquake struck the Apennines in Central Italy, causing the death of 299 people and destroying the majority of buildings in Amatrice, Arquata del Tronto, Accumoli, and other hamlets. The vast majority of the deaths, 238, were registered in Amatrice, the village where the study took place. Prior to the earthquake 2,464 people lived in Amatrice meaning that around 10% of the total population perished during the event. Further powerful shocks struck Central Italy on the 30^th^ of October 2016 (6.5 Mw with epicentre in Norcia, Umbria) and the 18^th^ of January 2017 (5.5 Mw with epicentre in Montereale, Abruzzo) further worsening the material damage but leading to no direct deaths in Amatrice and adjoining municipalities. Smaller earthquakes kept happening at the time the study was being conducted.

## 2. Material and methods

### 2.1. Participants

A sample of 139 directly-exposed survivors of the 2016–2017 Central Italy earthquakes was identified with the aid of the local municipality and health services who provided lists of people known to be residing in the area at the time data were being collected. The potential participants were contacted by telephone or face to face and given a standard introduction to the study. Participants were contacted individually in order to produce a sample that was representative of the population as whole (*i.e*. following age and gender distributions as per the 2016 census data of Amatrice (Istituto Nazionale di Statistica, 2016)). The only inclusion criterion was exposure to the earthquakes and residency in the affected region at the time of the research. Of the 139 individuals contacted, 127 agreed to participate (91% response rate).

### 2.2. Procedure

The UCL Research Ethics Committee approved the current study. The project was also approved by the health centre of Rieti, and by the local municipality, Comune di Amatrice. Data collection was conducted between the months of April and June 2017 in Amatrice, 3 months after the January 2017 earthquakes and 7 months after the August 2016 earthquakes. Participants gave written informed consent.

### 2.3. Measures

The Depression, Anxiety and Stress Scales-21 (DASS-21) had already been translated into Italian (Bottesi et al., 2015). All other questionnaires went through a back-translation procedure. The measures were first translated from English to Italian by the first author. The Italian translations were then given to a second translator fluent in both Italian and English who translated the measures back into English blind to the original versions. The original English version and the back-translated English version were then compared and discrepancies resolved.

#### 2.3.1. Independent variables

Prior to the blame and causal attribution questionnaires participants were asked to read a brief note with examples on the difference between the concepts of cause and blame (Shaver & Drown, 1986). Following the methodology of Lagnado and Channon (2008), each was rated using a single item. For causal attributions, participants were asked: ‘*How much do you think that the following entities have caused the earthquake damage?*’. Conversely, for blame attributions, participants were asked: ‘*How much do you think that the following entities are to blame for the earthquake damage?’*. Participants then rated on a Likert scale ranging from 0 (*minimum role in causation/not blameworthy at all*) to 9 (*maximum role in causation/extremely blameworthy*) the following entities: 1. Oneself 2. One’s family 3. The State 4. God 5. Chance 6. Nature 7. Building firms 8. Organized criminality, *i.e*. mafia 9. The municipality (*Comune*) 10. The community. The above entities were chosen on the basis of conversations with local community leaders. For consistency the focus of the attribution measures was kept on the various ‘actors’ rather than on consequences of the actors’ actions such as the quality of constructions (Ikizer, Karanci, & Doğulu, 2016; McClure, Allen, & Walkey, 2001). Participants were also given the chance to write down specific institutions or people that were not included in the previous list. Among these new entities were: the government engineering offices (*n* = 7), the municipality engineering offices (*n* = 5), the regional engineering offices (*n* = 3), and engineers and/or geometers in general (n = 3). The mayor, petrol extractions, nuclear experiments, the council estates administration, the Fine Arts administration, and specific people were each mentioned only once.

#### 2.3.2. Dependent variables

The PTSD Checklist for DSM-5 (PCL-5) is a 20-item self-report measure assessing the 20 DSM-5 symptoms for PTSD (Weathers et al., 2013), and respondents completed the questionnaire in reference to the earthquake. The self-report rating scale ranges from 0 (‘*not at all*’) to 4 (‘*extremely*’) focusing on how bothered the individual was by the symptoms in the last month. The PCL-5 has been shown to have high total internal reliability (α = .90) and acceptable to good internal reliability for the subscales (α range = .57–.78) (Sveen, Bondjers, & Willebrand, 2016). Internal reliability in the current study was high with a Cronbach’s α for the total score of .91.

The Depression, Anxiety and Stress Scale-21 (DASS-21) is a self-report scale of psychological distress, measuring rates of depression, anxiety, and stress (Lovibond & Lovibond, 1995). Individuals are asked to measure the severity of symptoms in the last week on a scale ranging from 0 (‘*not at all*’) to 3 (‘*most of the time*’). Internal reliability in the current study was high with α = .94 for the complete measure. The aggregated DASS-21 scores were used for all statistical analyses as an overall measure of psychological distress.

#### 2.3.3. Control variables: degree of earthquake exposure and resource loss

Following Fergusson, Horwood, Boden, and Mulder (2014), the Mercalli scale was used to measure direct exposure to the earthquakes. The Mercalli scale is a subjective tool to measure earthquake intensity on the basis of damage experienced by people and objects and the experience of shaking. Individuals were asked to identify the level with which they had experienced the strongest earthquake since, and including, the 24^th^ of August 2016.

The Conservation of Resources Evaluation (COR-E) is a self-report questionnaire devised by Hobfoll and Lilly (1993) in order to assess on a scale from 0 (‘*no loss’*) to 4 (*‘total loss’*) the extent of actual loss, threatened loss, or gain in resources in the previous 6 months. The questionnaire is based on the principles of the Conservation of Resources theory (Hobfoll, 1998) that has been shown to be particularly relevant following disasters (Sattler et al., 2006). The questionnaire includes 74 resources, spanning conditions (*e.g*. employment), personal characteristics (*e.g*. locus of control), energy (*e.g*. motivation), and objects (*e.g*. house). The COR-E was used in order to control for indirect trauma exposure in terms of resource loss. An aggregated total score for the sum of the threatened and actual loss items was used in all analyses.

Finally participants completed a series of demographic measures investigating gender, age, level of education, religious affiliation, and residency.

### 2.4. Data analysis

Psychopathology, earthquake exposure, and resource loss measures were square root transformed to achieve a normal distribution. Square root and logarithm transformations were attempted with the attribution data but, since they remained skewed, they were analysed using non-parametric tests. Boxplots were examined to identify potential multivariate outliers but none were found.

Descriptive statistics were conducted on all demographic, earthquake exposure, resource loss, and psychopathology variables. Independent sample t-tests were used to measure differences in PTSD and psychological distress according to gender, age, education, and religion. Wilcoxon signed-rank tests were used to assess the difference in mean ratings between causal and blame attributions for each entity. Significant differences would provide evidence that the two concepts were distinct in the minds of participants (H1). Associations between attributions and psychopathology variables, *i.e*. PCL-5 and DASS-21, were tested with Spearman zero-order correlations (H2). To adjust for multiple tests, we used the Benjamini-Hochberg approach to set a 5% false discovery rate. The Benjamini-Hochberg correction is designed to ensure that among those tests that are declared significant, the proportion of true null hypotheses is no more than 5% (Glickman, Rao, & Schultz, 2014). Finally, we investigated the association between attributions and psychopathology while controlling for potentially confounding variables using hierarchical multiple regression (H3). We first entered material loss, earthquake exposure, gender, age, and education in Step 1. In Step 2 we then included in the model the attribution ratings as additional correlates of psychopathology. To maintain an appropriate ratio of variables to sample size (Harrell, 2015) we only entered the attributions that had a significant relationship with psychopathology in the Spearman zero-order correlations. We ran the procedure separately for cause and blame attributions. Participants with missing data on individual variables were excluded from analyses involving these variables (11 participants had missing values on the COR-E, 8 participants had missing values on the blame attributions measure, 2 on the PCL-5, 1 on the Mercalli scale, and 1 on the cause attributions measure). All analyses were conducted using SPSS v. 23 (IBM Corp.).

## 3. Results

### 3.1. Participant characteristics

The majority of the participants were from Amatrice (*n* = 100) with a minority from Arquata del Tronto (*n* = 10), Accumoli (*n* = 6), and smaller adjoining municipalities (*n* = 11). The total sample was composed of 69 females (*M* age = 47.3, *SD *= 14.0, range 18–75) and 58 males (*M* age = 45.5, *SD = *18.5, range 18–75). Of the total sample, 80% of the participants (*n* = 102) identified as Catholic, with 9 participants identifying as agnostic, 8 as atheist, and 8 as ‘other’.

In terms of education, 25% of participants had not reached high school level, 55% had a high school diploma, 14% of participants had a university degree, and 3% had a post-graduate degree. The majority of participants reported either total (39%) or very substantial (40%) damage to property, with 10% of participants reporting medium damage, 8% minimum damage, and 3% no damage. In terms of human losses, 19% of individuals reported having lost close family members, that is, parents, children, or aunt/uncle/first-degree cousins, and all individuals reported losing close friends and acquaintances.

Participants reported that their median direct experience of shaking and damage was 9 out of 12, or ‘*disastrous*’ on the Mercalli scale measuring subjective earthquake intensity. The COR-E questionnaire measuring resource loss indicated that the highest reductions in resources were observed in ‘*housing that suits my needs*’ (*M* = 2.5, *SD* = 1.6), ‘*adequate home furnishing*’ (*M* = 2.0, *SD* = 1.7), and ‘*feeling that my life is peaceful*’ (*M* = 1.8, *SD* = 1.4). On a scale from 0 (no loss on any item) to 296 (maximum loss on every item), the mean score was 71 (*SD* = 46.95).

The mean score on the PCL-5 scale measuring PTSD symptoms was 29.7 (*SD* = 16.1). Fifty-one participants (40%) exceeded the clinical cut off score of 33 (U.S. Department of Veterans Affairs, 2018). The mean score on the DASS-21 scale measuring psychological distress was 19.8 (*SD* = 1.2). The transformed PCL-5 and DASS-21 scores were strongly correlated (*r* = .79, *p* < .01). Female participants displayed higher scores on the transformed PCL-5 (*t*(120) = −2.22, *p* < .05) but not on the transformed DASS-21 (*t*(122) = −.93, *p* > .05). No differences in psychopathology variables were found between religious and non-religious participants (*t*(123) = −.76, *p* = .447), between participants with higher education (*i.e*. university degree or above) and lower education (*t*(123) = .04, *p* = .963) and between younger (*i.e*. less than 40 years old) and older participants (*t*(122) = −.87, *p* = .387) following analyses with independent samples t-tests.

### 3.2. Distinction between attributions of cause and blame for the earthquake damage (H1)

The mean cause and blame attribution ratings for each entity are shown in Table 1. Attributions of cause and blame for the same entity were compared using the Wilcoxon signed-rank test. Results shown in Table 1 highlight how mean blame ratings were significantly greater than causal ratings for the State, building firms, the mafia, and the municipality. Conversely, causal ratings were greater than blame ratings for chance and nature. Attributions of cause and blame appeared to be conceptually distinct in the current sample.

**Table 1.** Mean cause and blame attributions for each entity in rank order of importance and comparisons between blame and cause attributions.

	Causal attributions		Blame attributions		Wilcoxon test	
	*M*	*SD*	*M*	*SD*	*Z*	*p*
Nature	7.5	2.7	5.5	4.0	−5.11	.000**
Building firms	4.6	3.3	5.6	2.9	−3.59	.000**
State	3.8	3.5	4.9	3.3	−4.17	.000**
Municipality	3.4	3.3	3.9	3.2	−2.18	.029*
Chance	2.8	3.5	2.3	3.3	−2.03	.042*
Mafia	2.0	3.0	2.5	3.2	−3.20	.001**
Community	2.0	2.7	1.8	2.6	−.42	.671
God	0.7	2.0	0.7	2.0	−.54	.589
Oneself	0.1	0.8	0.1	0.8	−.82	.412
One’s family	0.1	0.9	0.1	0.9	−1.06	.285

*p < .05, **p < .01 *Note*. For causal attributions, the scale anchors ranged from 0 = ‘*minimal role in causation*’ to 9 = ‘*maximum role in causation*’. For blame attributions from 0 = ‘*not blameworthy at all*’ to 9 = ‘*extremely blameworth*y’.

### 3.3. Attributions and psychopathology (H2)

Spearman zero-order correlations were calculated between the transformed psychopathology measures and attribution ratings for each entity. The unadjusted significance levels, shown in Table 2, highlight how blame and cause attributions towards God and chance were positively and significantly correlated with higher scores on both the PCL-5 and the DASS-21, whereas seeing oneself as a cause correlated with DASS-21 scores only. After applying a false discovery rate of 5% to control for multiple analyses through the Benjamini-Hochberg correction, all previously significant correlations between attributions and psychopathology remained significant except between blaming God and PCL-5 scores, and between seeing oneself as a cause and DASS-21 scores.

**Table 2.** Spearman correlations between cause and blame attributions to each entity and psychopathology.

	PCL-5	DASS-21
**Causal attributions**		
Nature	−.05	.00
Building firms	.00	−.05
State	.08	.07
Municipality	.02	.00
Chance	.28**	.32**
Mafia	.02	.07
Community	.00	−.05
God	.28**	.32**
Oneself	.09	.19*
One’s family	−.03	.05
**Blame attributions**		
Nature	.11	.11
Building firms	.03	.06
State	.05	.05
Municipality	−.03	.00
Chance	.28**	.33**
Mafia	−.03	.04
Community	−.04	−.06
God	.23*	.29**
Oneself	−.06	−.07
One’s family	−.12	−.15

*p < .05, **p < .01.

### 3.4. Associations between attributions and psychopathology while controlling for earthquake exposure, resource loss, gender, age, and education (H3)

Finally, we tested whether the associations between cause and blame attributions towards God and chance with psychopathology remained significant when controlling for demographic and exposure variables. Using hierarchical multiple regression we first investigated in Step 1 whether earthquake exposure, resource loss, gender, age, and education would be significantly associated with participants’ scores on the PCL-5 and DASS-21. We then entered in Step 2 the attribution variables that had significant relationships with psychopathology from the zero-order Spearman correlations (Table 2). The results separated between cause and blame attributions and between PCL-5 and DASS-21 scores are shown in Tables 3.1–3.4.

**Table 3.1.** Hierarchical multiple regression of causal attributions on PCL-5 scores.

	B	SE	β	*p*	R^2^	F
Step 1					0.12	3.03*
Material loss	0.10	0.40	0.26**	.008		
Earthquake exposure	0.48	0.41	0.11	.251		
Gender	0.43	0.27	0.14	.118		
Age	0.00	0.00	0.00	.975		
Education	−0.14	0.18	−0.07	.440		
Step 2					0.26	5.17**
Material loss	0.07	0.03	0.16	.068		
Earthquake exposure	0.57	0.38	0.13	.145		
Gender	0.57	0.26	0.19*	.029		
Age	0.00	0.00	0.03	.687		
Education	−0.13	0.17	−0.06	.453		
God (cause)	0.20	0.06	0.28**	.002		
Chance (cause)	0.08	0.03	0.19*	.035		

*p < .05, **p < .01.

**Table 3.2.** Hierarchical multiple regression of causal attributions on DASS-21 scores.

	B	SE	β	*p*	R^2^	F
Step 1					0.11	2.76*
Material loss	0.11	0.04	0.23*	.017		
Earthquake exposure	0.82	0.47	0.16	.089		
Gender	0.14	0.31	0.04	.638		
Age	0.00	0.01	0.04	.652		
Education	−0.20	0.21	−0.09	.336		
Step 2					0.27	4.79**
Material loss	0.06	0.04	0.13	.154		
Earthquake exposure	0.93	0.44	0.18*	.037		
Gender	0.32	0.29	0.09	.266		
Age	0.00	0.00	0.07	.415		
Education	−0.19	0.19	−0.08	.316		
God (cause)	0.19	0.08	0.24*	.016		
Chance (cause)	0.12	0.04	0.26**	.004		
Oneself (cause)	0.05	0.18	0.02	.783		

*p < .05, **p < .01.

**Table 3.3.** Hierarchical multiple regression of blame attributions on PCL-5 scores.

	B	SE	β	*p*	R^2^	F
Step 1					0.12	2.65*
Material loss	0.10	0.04	0.25*	.013		
Earthquake exposure	0.56	0.42	0.13	.193		
Gender	0.37	0.28	0.12	.195		
Age	0.00	0.00	0.00	.970		
Education	−0.13	0.19	−0.06	.494		
Step 2					0.23	4.23**
Material loss	0.07	0.04	0.18	.061		
Earthquake exposure	0.56	0.40	0.13	.162		
Gender	0.48	0.27	0.16	.080		
Age	0.00	0.00	0.04	.629		
Education	−0.12	0.18	−0.06	.480		
God (blame)	0.18	0.06	0.26**	.006		
Chance (blame)	0.08	0.04	0.18	.056		

*p < .05, **p < .01.

**Table 3.4.** Hierarchical multiple regression of blame attributions on DASS-21 scores.

	B	SE	β	*p*	R^2^	F
Step 1					0.11	2.54*
Material loss	0.11	0.04	0.23*	.023		
Earthquake exposure	0.82	0.47	0.16	.066		
Gender	0.14	0.31	0.04	.941		
Age	0.00	0.01	0.04	.796		
Education	−0.20	0.21	−0.09	.378		
Step 2					0.27	5.16**
Material loss	0.06	0.04	0.14	.125		
Earthquake exposure	0.91	0.43	0.19*	.039		
Gender	0.16	0.29	0.04	.579		
Age	0.00	0.00	0.08	.374		
Education	−0.18	0.19	−0.08	.350		
God (blame)	0.19	0.07	0.24**	.008		
Chance (blame)	0.13	0.04	0.27**	.003		

*p < .05, **p < .01.

Step 2 including cause attributions towards God and chance explained an additional 14% (ΔR^2^) of the variance in PCL-5 scores in comparison with Step 1. Female gender, cause attributions towards God and towards chance were significant correlates of PCL-5 scores in the final model. Due to gender being a significant correlate in Step 2, we conducted exploratory analyses to investigate differences in cause attributions towards God and chance between male and female participants using Mann-Whitney U tests but no differences were found (largest *Z* = −.296, *p* = .795)

Step 2 including cause attributions towards God, chance, and oneself explained an additional 16% (ΔR^2^) of the variance in DASS-21 scores in comparison with Step 1. Earthquake exposure, cause attributions towards God and towards chance were significant correlates of DASS-21 scores in the final model.

Step 2 including blame attributions towards God and chance explained an additional 11% (ΔR^2^) of the variance in PCL-5 scores in comparison with Step 1. Blame attributions towards God were significant correlates of PCL-5 scores in the final model. Blame attributions towards chance only approached significance (*p* = .056).

Step 2 including blame attributions towards God and chance explained an additional 16% (ΔR^2^) of the variance in DASS-21 scores in comparison with Step 1. Earthquake exposure, blame attributions towards God and towards chance were significant correlates of DASS-21 scores in the final model.

## 4. Discussion

An investigation among 127 survivors of the 2016–2017 Central Italy earthquakes provided support for the claim that individuals differentiate between blame and causal attributions following trauma and that specific attributions are associated with psychopathology. Ratings of cause and blame attributions differed from one another on several entities. In particular, attributions to the State, building firms, the mafia, and the municipality were associated with significantly greater ratings of blame than cause attributions, whereas attributions to nature and chance were associated with significantly greater cause than blame attributions.

Cause and blame attributions towards God and chance were significantly correlated with psychopathology to a similar degree, the findings remaining significant when controlling for degree of earthquake exposure, resource loss, gender, age, and education. In summary, therefore, the findings supported our three hypotheses on the distinction between cause and blame attributions (H1), and on the positive relationship between attributions to God and psychopathology (H2), even when controlling for resource loss, earthquake exposure, and demographic variables (H3). Additionally, overall the evidence did not suggest that gender was a risk factor for psychopathology in this sample. There was one exception: gender was a significant correlate of PCL-5 scores in the regression model including cause attributions towards God and chance (Table 3.1). Given the overall pattern of results, however, this finding should be treated with caution.

The distinction found between cause and blame is in line with findings from laboratory studies demonstrating the difference between the two concepts (Critchlow, 1985), while also supporting previous theories on the difference between cause and blame (Shaver & Drown, 1986). The distinction is also consistent with the findings of the only previous study that assessed the distinction between attributions of cause and blame following a traumatic event (Brewin, 1984). The higher level of blame attributions towards the State, building firms, the municipality, and the mafia, as opposed to the lower levels of blame of nature and chance, might be explained by the relative importance of intentionality, possible knowledge, foreseeability, and morally questionable motives in characterizing blame versus cause attributions. Several laboratory manipulations have indeed highlighted the role of intentionality and foreseeability (Lagnado & Channon, 2008) in blame judgements. Furthermore, morally questionable motives, such as the widespread belief held by the community concerning the thirst for profit of building firms or of the State, have also been shown to exacerbate blame judgements (Alicke, 1992). Intentionality, knowledge, foreseeability, and motives have indeed all been identified as core elements within contemporary theoretical models of blame (Alicke, Mandel, Hilton, Gerstenberg, & Lagnado, 2015).

Attributions to God following natural disasters can be common among survivors (Stephens, Fryberg, Markus, & Hamedani, 2013). Nonetheless, little research has yet investigated the relationship between such attributions and psychopathology. The limited existing evidence does however support a positive association between attributions to God and psychopathology following an earthquake in Turkey (Sezgin & Punamäki, 2012), an earthquake in Kashmir (Feder et al., 2013), a tornado in the US (Lack & Sullivan, 2008), and a flood in the US (Smith et al., 2000). The only study that assessed chance attributions following earthquakes in Iceland also found a positive association between luck attributions and psychopathology (Bödvarsdóttir & Elklit, 2004).

Several factors may underpin these associations. We hypothesize that attributions to God reflect a broader shattering of world-view assumptions (Janoff-Bulman, 1989) and the challenging of pre-trauma inner schemas of the world (Horowitz, Wilner, Kaltreider, & Alvarez, 1980). A drastic change in world-view may result from the conclusion that what was likely to be perceived as a benign entity such as God could potentially punish humans in such a harsh way. A hallmark of PTSD has long been believed to be a shattering of assumptions, including religious assumptions (Falsetti, Resick, & Davis, 2003), and consequent dissonance (Elwood, Hahn, Olatunji, & Williams, 2009). Additionally, decreased participation and engagement with religious activities resulting from spiritual disillusionment may mediate the positive association between attributions to God and psychopathology. The community involvement and ritual aspects of religion have previously been found to foster positive coping mechanisms following disasters (Park, 2016), for example in the aftermath of the L’Aquila earthquake (Stratta et al., 2013). The low levels of cause and blame attributions towards God are likely to reflect the Catholic belief that humans are gifted with agency and free-will leading the majority of participants to absolve God from the responsibility of the earthquake damage (Massazza, Brewin, & Joffe, 2019).

If the ultimate aim of attributions is that of ‘*encouraging and maintaining effective exercise of control in the world*’ (Kelley, 1971, p. 22), chance attributions are likely to fail at this task. Chance attributions could be conceptualized as signs of an inability to make meaning out of the traumatic experience (Frankl, 1962) and, consequently, of maintaining control over the situation (Rothbaum, Weisz, & Snyder, 1982). Attributing an event to chance may imply a collapse into the unpredictable and the uncontrollable.

The main limitation of the current study is the use of a purposive sample. Kessler, Keane, Ursano, Mokdad, and Zaslavsky (2008) argued for the use of probability household sampling among survivors of natural disasters. However, the extensive material damage caused by the earthquake meant that virtually no participant had an intact house. Participants lived in temporary housing such as containers and campers which were not documented by the municipality making randomisation impossible. Due to the geographical structure of Amatrice, many hamlets remained isolated in the mountains and were often physically hard to reach due to rubble still blocking many roads. Additionally, parts of the population remained highly mobile, moving between different temporary housing solutions, *e.g*. other family members’ houses in towns close to Amatrice or hotels provided by the State on the coast. Official temporary housing provided by the State was not ready yet.

All these factors meant that systematic probability sampling was not possible. Informal lists of residents provided by the health centre and municipality were the most effective and reliable source of recruitment and identification of potential participants. The local municipality recorded 1000 people living in Amatrice at the time of data collection which means that more than 10% of the population was surveyed therefore reducing the possibility of systematic bias in the sample. Additionally, the gender and age distributions of the current sample broadly reflected the official demographics of Amatrice’s population as a whole (Istituto Nazionale di Statistica, 2016).

Another limitation concerns the correlational nature of the study. This hinders causal inference concerning the directionality of the association between attributions and psychopathology. Furthermore, social desirability might limit the willingness of participants to engage in blame attributions (Thompson, 1985).

The current findings have potential implications for the treatment of psychopathology among disaster survivors. Guidelines for the treatment of PTSD (Foa, Keane, & Friedman, 2009) have recommended the use of attribution retraining, that is, a form of cognitive restructuring aimed at providing patients with more realistic and adaptive attributions (Försterling, 1985). Clinicians working with disaster victims might therefore look out for attributions towards God or chance as potential indicators of increased distress. The inclusion of spiritual leaders and local informants within the mental health response to disasters might also be a helpful addition in highly religious communities such as the one surveyed here. However further research using longitudinal and probability-sampling cohorts will be necessary to confirm the current findings.

Future research might also investigate how and why certain attributions are formed following trauma, a topic that has been under-researched (Massad & Hulsey, 2006). More attention should also be paid to the emotional aspect of attributions, particularly due to the significant affective components of blame (Alicke et al., 2015). Furthermore, future researchers might want to investigate the association between attributions and other forms of psychopathology which have been found to be common following disasters, such as substance use disorders (Katz, Pellegrino, Pandya, Ng, & DeLisi, 2002).

As the first study of the relationship between psychopathology and cause/blame attributions among earthquake survivors, the current study actively contributes to the understanding of the role played by attributions following trauma. In particular, it identifies that high levels of PTSD and psychological distress symptoms are associated, in the community under study, with attributions to God and to chance. The current findings are relevant to understanding how the DSM-5 symptom D3 for PTSD, *i.e*. ‘*persistent, distorted cognitions about the cause or consequences of the traumatic event(s) that lead the individual to blame himself/herself or others*’ (APA, 2013, p. 272), may be expressed in different contexts. In particular, we identified that following disaster attributions of cause and blame towards certain entities, but not towards others, were associated with higher levels of psychopathology. It will be important to investigate whether similar patterns of attributions are observed following other complex emergencies in order to uncover the fractures that disasters, and trauma more generally, can create in communities’ worldviews.

## References

[cit0001] Alicke M. D. (1992). Culpable causation. *Journal of Personality and Social Psychology*, 63, 368–11.

[cit0002] Alicke M. D. (2000). Culpable control and the psychology of blame. *Psychological Bulletin*, 126, 556–574. 10900996 10.1037/0033-2909.126.4.556

[cit0003] Alicke M. D., Mandel D. R., Hilton D. J., Gerstenberg T., & Lagnado D. A. (2015). Causal conceptions in social explanation and moral evaluation: A historical tour. *Perspectives on Psychological Science*, 10, 790–812. 26581736 10.1177/1745691615601888

[cit0004] American Psychiatric Association (2013). *Diagnostic and statistical manual of mental disorders* (5th ed.). Washington, DC: American Psychiatric Association.

[cit0005] Aten J. D., Smith W. R., Davis E. B., Van Tongeren D. R., Hook J. N., Davis D. E., … Hill P. C. (2019). The psychological study of religion and spirituality in a disaster context: A systematic review. *Psychological Trauma: Theory, Research, Practice, and Policy*. 10.1037/tra0000431 30730187

[cit0006] Banford A. J., Wickrama T., & Ketring S. (2014). Physical health problem intrusions linking religious attributions to marital satisfaction in survivors of the 2004 tsunami. *Journal of Geography and Natural Disasters*, 4, 118.

[cit0007] Bödvarsdóttir I., & Elklit A. (2004). Psychological reactions in Icelandic earthquake survivors. *Scandinavian Journal of Psychology*, 45, 3–13. 15016274 10.1111/j.1467-9450.2004.00373.x

[cit0008] Bottesi G., Ghisi M., Altoè G., Conforti E., Melli G., & Sica C. (2015). The Italian version of the depression anxiety stress scale-21: Factor structure and psychometric properties on community and clinical samples. *Comprehensive Psychiatry*, 60, 170–181. 25933937 10.1016/j.comppsych.2015.04.005

[cit0009] Brewin C. R. (1984). Attributions for industrial accidents: Their relationship to rehabilitation outcome. *Journal of Social and Clinical Psychology*, 2, 156–164.

[cit0010] Critchlow B. (1985). The blame in the bottle: Attributions about drunken behavior. *Personality and Social Psychology Bulletin*, 11, 258–274.

[cit0011] Dai W., Chen L., Zhiwei L., Yan L., Wang J., & Liu A. (2016). The incidence of post- traumatic stress disorder among survivors after earthquakes: A systematic review and meta-analysis. *BMC Psychiatry*, 16, 188. 27267874 10.1186/s12888-016-0891-9 PMC4895994

[cit0012] Dollinger S. J. (1986). The need for meaning following disaster: Attributions and emotional upset. *Personality and Social Psychology Bulletin*, 12, 300–310.

[cit0013] Elwood L. S., Hahn K. S., Olatunji B. O., & Williams N. L. (2009). Cognitive vulnerabilities to the development of PTSD: A review of four vulnerabilities and the proposal of an integrative vulnerability model. *Clinical Psychology Review*, 29, 87–100. 19008028 10.1016/j.cpr.2008.10.002

[cit0014] Falsetti S. A., Resick P. A., & Davis J. L. (2003). Changes in religious beliefs following trauma. *Journal of Traumatic Stress*, 16, 391–398. 12895022 10.1023/A:1024422220163

[cit0015] Feder A., Ahmad S., Lee E. J., Morgan J. E., Singh R., Smith B. W., … Charney D. S. (2013). Coping and PTSD symptoms in Pakistani earthquake survivors: Purpose in life, religious coping and social support. *Journal of Affective Disorders*, 147, 156–163. 23196198 10.1016/j.jad.2012.10.027

[cit0016] Feiring C., & Cleland C. M. (2007). Childhood sexual abuse and abuse-specific attributions of blame over six years following discovery. *Child Abuse and Neglect*, 31(11–12), 1169–1186. 18023871 10.1016/j.chiabu.2007.03.020 PMC2149908

[cit0017] Fergusson D. M., Horwood J., Boden J. M., & Mulder R. T. (2014). Impact of a major disaster on the mental health of a well-studied cohort. *JAMA Psychiatry (Chicago, IL)*, 71, 1025–1031. 10.1001/jamapsychiatry.2014.652 25028897

[cit0018] Foa E. B., Keane T. M., & Friedman M. J. (2009). *Effective treatment for PTSD: Practice guidelines from the international society for traumatic stress studies* (2nd ed.). New York: Guilford Press.

[cit0019] Försterling F. (1985). Attributional retraining: A review. *Psychological Bulletin*, 98, 495–512. 3909186

[cit0020] Frankl V. (1962). *Man’s search for meaning: An introduction to logotherapy*. Boston, MA: Beacon.

[cit0021] Glickman M. E., Rao S. R., & Schultz M. R. (2014). False discovery rate control is a recommended alternative to Bonferroni-type adjustments in health studies. *Journal of Clinical Epidemiology*, 67, 850–857. 24831050 10.1016/j.jclinepi.2014.03.012

[cit0022] Greening L., Stoppelbein L., & Docter R. (2002). The mediating effects of attributional style and event-specific attributions of postdisaster adjustment. *Cognitive Therapy and Research*, 26, 261–274.

[cit0023] Hall S., French D. P., & Marteau M. (2003). Causal attributions following serious unexpected negative events: A systematic review. *Journal of Social and Clinical Psychology*, 22, 515–536.

[cit0024] Harrell F. E. (2015). *Regression modelling strategies: With applications to linear models, logistic and ordinal regression, and survival analysis* (2nd ed.). New York: Springer-Verlag.

[cit0025] Hobfoll S. E. (1998). *Stress, culture and community*. New York: Plenum Press.

[cit0026] Hobfoll S. E., & Lilly S. (1993). Resource conservation as a strategy for community psychology. *Journal of Community Psychology*, 21, 128–148.

[cit0027] Horowitz M. K., Wilner N., Kaltreider N., & Alvarez N. (1980). Signs and symptoms of posttraumatic stress disorder. *Archives of General Psychiatry*, 37, 85–92. 7352843 10.1001/archpsyc.1980.01780140087010

[cit0028] Ikizer G., Karanci A. N., & Doğulu C. (2016). Exploring factors associated with psychological resilience among earthquake survivors from Turkey. *Journal of Loss and Trauma*, 21(5), 348–398.

[cit0029] Istituto Nazionale di Statistica (2016). Popolazione per età, sesso e stato civile 2016 (Amatrice). Retrieved from https://www.tuttitalia.it/lazio/82-amatrice/statistiche/popolazione-eta-sesso-stato-civile-2016/

[cit0030] Janoff-Bulman R. (1989). Assumptive worlds and the stress of traumatic events: Applications of the schema construct. *Social Cognition*, 7, 113–136.

[cit0031] Jeney-Gammon P., Daugherty T. K., Finch A. J., Belter R. W., & Foster K. Y. (1993). Children’s coping styles and report of depressive symptoms following a natural disaster. *Journal of Genetic Psychology*, 154, 259–267. 8366334 10.1080/00221325.1993.9914739

[cit0032] Joseph S., Yule W., & Williams R. (1993). Post-traumatic stress: Attributional aspects. *Journal of Traumatic Stress*, 6(4), 501–513.

[cit0033] Katz C. L., Pellegrino L., Pandya A., Ng A., & DeLisi L. E. (2002). Research on psychiatric outcomes and interventions subsequent to disasters: A review of the literature. *Psychiatry Research*, 110, 201–217. 12127471 10.1016/s0165-1781(02)00110-5

[cit0034] Kelley H. H. (1971). *Attributions in social interactions*. Morristown, NJ: General Learning Press.

[cit0035] Kessler R. C., Keane T. M., Ursano R. J., Mokdad A., & Zaslavsky A. M. (2008). Sample and design considerations in post-disaster mental health needs assessment tracking surveys. *International Journal of Methods in Psychiatric Research*, 17, S6–S20. 19035440 10.1002/mpr.269 PMC3081100

[cit0036] Lack C. W., & Sullivan M. A. (2008). Attributions, coping, and exposure as predictors of long-term posttraumatic distress in tornado-exposed children. *Journal of Loss and Trauma*, 13, 72–84.

[cit0037] Lagnado D. A., & Channon S. (2008). Judgments of cause and blame: The effects of intentionality and foreseeability. *Cognition*, 108, 754–770. 18706537 10.1016/j.cognition.2008.06.009

[cit0038] Levy B. R., Slade M. D., & Ranasinghe P. (2009). Causal thinking after a tsunami wave: Karma beliefs, pessimistic explanatory style and health among Sri Lankan survivors. *Journal of Religion and Health*, 48, 38–45. 19229624 10.1007/s10943-008-9162-5

[cit0039] Lovibond S. H., & Lovibond P. F. (1995). *Manual for the Depression, Anxiety, Stress Scales (DASS)*. New South Wales: Psychology Foundation Monograph.

[cit0040] Malle B. F., Guglielmo S., & Monroe A. E. (2014). A theory of blame. *Psychological Inquiry*, 25, 147–186.

[cit0041] Massad P. M., & Hulsey T. L. (2006). Causal attributions in posttraumatic stress disorder: Implications for clinical research and practice. *Psychotherapy: Theory, Research, Practice, Training*, 43, 201–215. 10.1037/0033-3204.43.2.201 22122038

[cit0042] Massazza A., Brewin C. R., & Joffe H. (2019). The nature of “natural disasters”: Survivors’ explanations of earthquake damage. *International Journal of Disaster Risk Science*.

[cit0043] McCaughey B. G., Hoffman K. J., & Llewellyn C. H. (1995). Chapter 6: The human experience of earthquakes In Ursano R. J., McCaughey B. G., & Fullerton C. S. (Eds.), *Individual and community responses to trauma and disaster: The structure of human chaos* (pp. 136–154). Cambridge: Cambridge University Press.

[cit0044] McClure J., Allen M. W., & Walkey F. (2001). Countering fatalism: Causal information in news reports affects judgements about earthquake damage. *Basic and Applied Social Psychology*, 23(2), 109–121.

[cit0045] Olff M. (2017). Sex and gender differences in post-traumatic stress disorder: An update. *European Journal of Psychotraumatology*, 8(sup4), 1351204.

[cit0046] Park C. L. (2016). Meaning making in the context of disaster. *Journal of Clinical Psychology*, 72, 1234–1246. 26900868 10.1002/jclp.22270

[cit0047] Rothbaum F., Weisz J., & Snyder S. (1982). Changing the world and changing the self: A two process model of perceived control. *Journal of Personality and Social Psychology*, 42, 5–37.

[cit0048] Sattler D. N., Glower de Alvarado A. M., Blandon de Castro N. B., van Male R., Zetino A. M., & Vega R. (2006). El Salvador earthquakes: Relationship among acute stress disorder symptoms, depression, traumatic event exposure, and resource loss. *Journal of Traumatic Stress*, 19, 879–893. 17195987 10.1002/jts.20174

[cit0049] Sezgin U., & Punamäki R. L. (2012). Earthquake trauma and causal explanation associating with PTSD and other psychiatric disorders among South East Anatolian women. *Journal of Affective Disorders*, 141, 432–440. 22464217 10.1016/j.jad.2012.03.005

[cit0050] Shaver K. G., & Drown D. (1986). On causality, responsibility, and self-blame: A theoretical note. *Journal of Personality and Social Psychology*, 50, 697–702. 3712221 10.1037//0022-3514.50.4.697

[cit0051] Smith B. W., Pargament K. I., Brant C., & Oliver J. M. (2000). Noah revisited: Religious coping by church members and the impact of the 1993 midwest flood. *Journal of Community Psychology*, 28, 169–186.

[cit0052] Solomon S. D., Regier D. A., & Burke J. D. (1989). Role of perceived control in coping with disaster. *Journal of Social and Clinical Psychology*, 8, 376–392.

[cit0053] Stephens N. M., Fryberg S. A., Markus H. R., & Hamedani M. G. (2013). Who explains Hurricane Katrina and the Chilean earthquake as an act of God? The experience of extreme hardship predicts religious meaning-making. *Journal of Cross-cultural Psychology*, 44, 606–619.

[cit0054] Stratta P., Capanna C., Riccardi I., Perugi G., Toni C., Dell’Osso L., & Rossi A. (2013). Spirituality and religiosity in the aftermath of a natural catastrophe in Italy. *Journal of Religion and Health*, 52, 1029–1037. 22395757 10.1007/s10943-012-9591-z

[cit0055] Stratta P., Rossetti M. C., Di Michele V., & Rossi A. (2016). Gli effetti sulla salute del sisma dell’Aquila. *Epidemiologia E Prevenzione*, 40, 22–32. 10.19191/EP16.2S1.P022.044 27291204

[cit0056] Sveen J., Bondjers K., & Willebrand M. (2016). Psychometric properties of the PTSD checklist for DSM-5: A pilot study. *European Journal of Psychotraumatology*, 7, 30165. 27098450 10.3402/ejpt.v7.30165 PMC4838990

[cit0057] Thompson S. C. (1985). Finding positive meaning in a stressful event and coping. *Basic and Applied Social Psychology*, 6, 279–295.

[cit0058] U.S. Department of Veterans Affairs (2018, 2 19). PTSD Checklist for DSM-5 (PCL-5). Retrieved from https://www.ptsd.va.gov/professional/assessment/adult-sr/ptsd-checklist.asp

[cit0059] Weathers F., Litz B., Keane T., Palmieri T., Marx B. P., & Shnurr P. (2013). The PTSD Checklist for DMS-5 (PCL-5). Retrieved from https://www.ptsd.va.gov/professional/assessment/documents/PCL-5_Standard.pdf

